# Multidimensional assessment of cervical spondylotic myelopathy patients. Usefulness of a comprehensive score system

**DOI:** 10.1007/s10072-020-04691-0

**Published:** 2020-09-03

**Authors:** Fabio Pilato, Rosalinda Calandrelli, Marisa Distefano, Francesco Ciro Tamburrelli

**Affiliations:** 1 UOC Neurologia, Dipartimento di scienze dell’invecchiamento, neurologiche, ortopediche e della testa-collo, Fondazione Policlinico Universitario A. Gemelli – IRCCS, 00168 Rome, Italy; 2 UOC Radiologia e Neuroradiologia, Dipartimento di diagnostica per immagini, radioterapia oncologica ed ematologia, Fondazione Policlinico Universitario A. Gemelli – IRCCS, Rome, Italy; 3 UOC Neurologia e UTN, Ospedale Belcolle, Strada Sammartinese, 01100 Viterbo, Italy; 4 UOC Chirurgia Vertebrale, Dipartimento di scienze dell’invecchiamento, neurologiche, ortopediche e della testa-collo, Fondazione Policlinico Universitario A. Gemelli – IRCCS, Rome, Italy; 5 Istituto di Ortopedia, Università Cattolica del Sacro Cuore, Rome, Italy

**Keywords:** Magnetic resonance imaging, Cervical spondylotic myelopathy, Prognosis, Motor-evoked potentials, Myelopathy, Personalized medicine

## Abstract

**Objective:**

Cervical spondylotic myelopathy (CSM) is caused by cervical spine degeneration and surgery may be beneficial, but selection for surgery might be challenging. We performed a multimodal analysis to assess predicting factors that may be useful to help surgeons in this choice.

**Patients and methods:**

We retrospectively evaluated clinical, motor evoked potentials (MEP), and MRI data of patients who undergone surgery for CSM. Seventy-six consecutive patients (46 males) were enrolled. The median age was 65.5 [IQR: 57–71] years, and the duration of symptoms was 11 [8–13] months. A multivariate analysis in order to assess predictors of outcome and ROC curve analysis were performed.

**Results:**

Thirty patients (M:18, 39.5%) gained 6 or more points on mJOA and they were collected in good recovery group, whereas 46 patients (60.5%, M:28) showed a fair recovery. We developed a comprehensive score system (CSS) taking into account clinical, neurophysiological, and neuroradiological data. ROC curve analysis was performed to determine the discriminative power of four models derived from the multivariate logistic regression analysis for predictors of good outcome considering only clinical variables, MRI variables, and MEP variables or considering the comprehensive model, demonstrating a good accuracy of CSS model to predict outcome.

**Conclusion:**

This study demonstrates that CSS model taking into consideration functional assessment by mJOA score, neurologic evaluation, cervical MRI, and MEP may be a feasible method to predict outcome in patients candidate to surgery, supporting surgeon’s decisions both for those patients candidate to surgery and for patients in whom a “wait and see” approach could be proposed.

**Electronic supplementary material:**

The online version of this article (10.1007/s10072-020-04691-0) contains supplementary material, which is available to authorized users.

## Introduction

Cervical spondylotic myelopathy (CSM) is a neurological disorder caused by the degeneration of the spine and resultant spinal cord compression [2]. It is an ongoing process and patients present at various clinical stages of severity, ranging from no clinical sign to severe neurological impairment [6, 24]. It has become a prevalent cause of spinal cord dysfunction in aging population worldwide leading to severe physical disability. Due to the benign nature and insidious development of CSM, it had been less recognized until advanced medical technologies such as magnetic resonance imaging (MRI) and electrophysiological examinations became prevalent in recent decades. Surgery is a widely accepted treatment for severe CSM with neurological complications. However, for asymptomatic or mildly symptomatic patients with CSM, the optimal management remains debated and surgery is sometimes controversial [4].

MRI is a gold standard for CSM because it shows not only the anatomy and location of compression in the cervical spine but also intramedullary signal intensity changes in the cervical spinal cord suggestive of spinal cord lesion [23]. On the other hand, MRI reveals only morphology and not functionality of the spinal cord and MRI-driven decisions about the timing for surgery are controversial [20]. Neurophysiological techniques such as somatosensory-evoked potentials (SEP) [18] and motor-evoked potentials (MEP) may be useful tools to evaluate functionality of the cervical spinal cord [3, 8, 20], and a prognostic role in CSM patients referred to surgery was reported [3, 19, 20].

Previous studies evaluated whether clinical assessment, MRI, or MEP independently could have predicting value on surgical outcomes [3, 17, 20], but conclusive results are still lacking even if correlations between presurgical MRI findings, neurophysiological assessment, and surgical outcomes were found [16, 20].

We evaluated clinical, neurophysiological, and neuroradiological data, and results linked with better outcome were used to develop a comprehensive model.

## Patients and methods

We retrospectively investigated medical records, MEP, and MRI scans of patients who underwent surgery for CSM between January 2004 and December 2014 referred to our university hospital.

Diagnoses of CSM were made on the basis of the results of neurological examinations, neurophysiological tests, and diagnostic imaging using various techniques including MRI. The exclusion criteria were history of any spine surgery; thoracic or lumbar spinal diseases based on relevant clinical symptoms; and imaging tests and history of rheumatoid arthritis, cerebral palsy, or tumors.

After database revision, 76 consecutive patients (46 males and 30 females) were enrolled. All patients enrolled in this study had a minimum 1-year follow-up time, a complete neurological and neurophysiological assessment and underwent preoperative cervical MRI.

In all patients, the cervical cord was compressed at some point between the C3/4 and the C6/7 levels.

Surgical approaches and operated levels varied by case and were chosen based on both the patient’s conditions and the surgeon’s clinical experience.

Clinical assessment and 18-point modified Japanese Orthopedic Association (mJOA) scores were recorded [27]. Neurophysiological evaluation with MEP was performed in preoperative clinical evaluation period, and postoperative outcomes (6–12 months) were assessed by on-site follow-up visits.

Seventy-six patients with MRI documented CSM (50), cervical posterior longitudinal ligament ossification (4), and disc disease (22) underwent instrumented laminectomy (56), anterior cervical discectomy and fusion (14), or open door laminoplasty (6).

The median age was 65.5 [57–71] years, and the median duration of symptoms was 11 [8–13] months.

According to postsurgical mJOA score, patients were divided in 2 groups: good recovery and fair recovery groups. As previously measured, good recovery was defined as a complete recovery or improvements of at least 30% (six or more points) in follow-up mJOA evaluation compared with presurgical evaluation [3], whereas patients with suboptimal recovery were defined as less than 30% (five or lower points) in the comparative assessment. No patient showed worse scores in follow-up evaluation compared with presurgical evaluation, and no surgical complication was recorded.

### Clinical evaluation

All patients underwent to a complete neurological examination, functional assessment, and neurophysiological evaluation both before and after surgery.

The clinical severity of neurologic function before and after surgery was assessed by the mJOA scoring system [27], and pyramidal signs such as Babinski’s sign were recorded. Thirty patients (18 males and 12 females) showed a good recovery in follow-up visit, and 46 patients (28 males and 18 females) showed a fair recovery.

### Magnetic resonance imaging

**Fig. 1:**
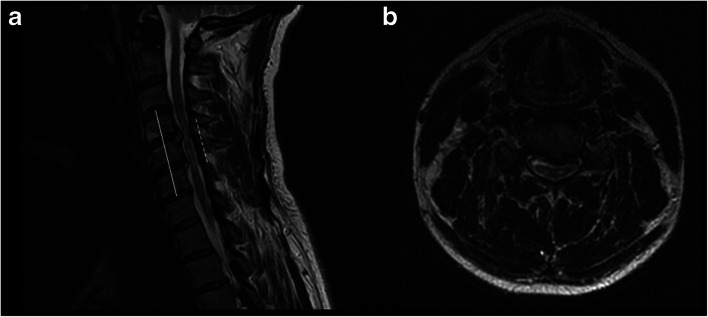
MRI of a patient with central canal stenosis and myelopathy. Sagittal T2wi MR shows severe central canal stenosis extending from C4 to C6 levels with reduction of subarachnoid spaces. The central cord hyperintensity is documented to the maximum spinal cord compression level extending from C4 to C5 (**a**). Gray matter intramedullary T2 hyperintensity is well shown on axial T2 images (**b**)

All patients underwent cervical MRI according to a standard protocol on a 1.5 T Signa Unit (General Electric Healthcare; Milwaukee, WI, USA) with a standard coil. MRI consisted of T1-weighted and T2-weighted images (T2wi) in the axial and sagittal planes. Being the aim of the study mainly focused on the relationship among clinical aspects, diagnostic imaging, and neurophysiological investigation, MRI evaluation was limited to the following aspects: presence of narrowing of the cervical spinal canal, presence of signal changes inside the cord, and proximo-distal extension of the cord compression (Fig. 1). Extension of cervical stenosis was considered the level between C3 and C7 in which CSF space was obliterated around the spinal cord and consequent compression of the spinal cord compared with spinal canal above C3 or below C7 level. Cervical lesion was defined as intramedullary hyperintensity on T2wi on sagittal and axial imaging [26]. A visual analysis at the compressed and reference levels, on sagittal and axial T2wi, was performed by an experienced neuroradiologist blinded about group assignment.

### Motor-evoked potentials

A Magstim 200 stimulator (Magstim, Whitland, UK) was used to deliver transcranial and paravertebral stimuli through a 120-mm circular coil. MEP was recorded from the biceps, abductor digiti minimi, and tibialis anterior muscles in all patients. Cortical stimulation was performed at maximum output of the stimulator during tonic activation at about 20% of maximum voluntary contraction of the tested muscle following protocol previously described [8]. Paravertebral stimulation was performed at rest using a magnetic stimulus intensity of 60% of maximum magnetic stimulator output. Central motor conduction time (CMCT) was calculated by subtracting the peripheral conduction time from spinal cord to muscles from the latency of responses evoked by cortical stimulation, and results were considered abnormal if CMCT measurement, after superimposing the responses, a reproducible onset latency measured above reference values [8, 21].

### Statistical analysis

Demographics and clinical characteristics were compared between subjects with good and fair recovery defined as gaining > 6 points at post-mJOA evaluation. For continuous measures, means and SD, medians, and interquartile ranges [IQR] are presented and *p* values calculated with a two-tailed *t* test for Gaussian continuous variables and the Mann-Whitney *U* or Kruskal-Wallis test for non-Gaussian continuous variables. Normality distribution was tested with Shapiro Wilk’s test. For categorical measures, frequencies and percentages are presented and *p* values calculated with a χ2 or a two-tailed Fisher’s exact test as appropriate.

A multivariate analysis was performed using a logistic regression model with favorable outcome as dependent variable; except for age and sex, only variables with *p* value less than 0.1 at univariate analysis were included into the multivariate models.

Receiver operator characteristic (ROC) curve analysis was performed to determine the discriminative power (area under the curve [AUC]) of four models derived from the multivariate logistic regression analysis for good outcome: considering only clinical variables, neuroradiological variables, and MEP variables separately or considering the comprehensive model (CSS).

Since we excluded patients with missing essential data from our analysis, we did not impute for missing data. Statistical significance threshold was set at *p* = 0.05.

Statistics were performed by means of the Statistical Package for Social Science (SPSS®) software version 25.

## Results

Table 1 summarizes the demographics, clinical characteristics and neuroradiological and MEP results of enrolled patients.

**Table 1:** Demographics, clinical characteristics, and neuroradiological and MEP results of enrolled patients

Demographic characteristics	All patient	Good recovery	Fair recovery	*p* value
Gender N. of patients (%)	46 M, (60.5%) 30 F, (39.5%)	18 M, (60%) 12 F, (40%)	28 M, (60.9%) 18 F, (39.1%)	0.940
Age (years) Median [IQR]	65.5 [57–71]	67 [63–68]	60 [54–71]	0.180
Duration of symptoms (months) Median [IQR]	11 [8–13]	8.5 [7–13]	12 [9–13]	0.068
Preoperative mJOA score Median [IQR]	10 [9–12]	10 [8–10]	11 [9–12]	*0.025*
Postoperative mJOA score Median [IQR]	16 [14–17]	16 [15–17]	14 [13–16]	*0.002*
MRI features				
Preoperative length of MRI lesion (mm) Median [IQR]	12 [6–16]	6 [3–9]	12 [12–16]	*<0.001*
Neurological signs				
Babinski’s sign, N. of patients, (%)	46 (60.5%)	13 (43.3%)	33 (71.7%)	*0.013*
MEP Pre-surgery				
CMCT (ms) Median [IQR]				
Biceps (n.v. 7.6)	6.4 [5.6–6.9]	6.5 [5.7–7.6]	6.3 [5.6–6.7]	0.093
Abductor digiti minimi (n.v. 7.7)	11.6 [9.4–13.8]	12.2 [10.3–14.4]	10.8 [8.8–13.8]	0.089
Tibialis anterior (n.v. 17.1)	22.6 [19.9–24.8]	23.4 [21.2–25.5]	21.6 [18.2–23.9]	0.079
MEP Post-surgery				
Biceps (n.v. 7.6)	6.4 [5.8–6.8]	6.3 [5.8–6.6]	6.4 [5.8–6.8]	0.360
Abductor digiti minimi (n.v. 7.7)	11.6 [9.7–13.2]	11.6 [9.7–13.1]	11.4 [9.3–14.6]	0.734
Tibialis anterior (n.v. 17.1)	21.4 [18.9–24]	21.9 [19.7–24]	20.8 [18.8–23.2]	0.372

CMCT, central motor conduction time; ms, milliseconds; mm, millimeters; mJOA, modified Japanese Orthopedic Association; n.v., normal value

According to postsurgical evaluation, 30 patients (M: 18, 39.5%) gained 6 or more points on mJOA and they were collected in good recovery group, whereas 46 patients (60.5%, M: 28) showed a fair recovery gaining 5 or less points. Presurgical assessment did not show differences in all baseline demographic characteristics between groups (*p* > 0.05), but median mJOA score was worse in good recovery group 10 [8–10] vs 11 [9–12] (*p* = 0.025), and more patients in fair group showed Babinski’s sign (71.7% vs 43.3%) (*p* = 0.013). In enrolled patients, median duration of symptoms was 11 months [8–13] (*p* > 0.05). Presurgical MRI evaluation showed a significant difference between groups in length of the lesion on T2wi showing longer lesions in fair recovery group 12 mm [12–16] compared with 6 mm [3–9] in good recovery group (*p* < 0.001). Presurgical MEP between groups did not show a significant difference (*p* > 0.05).

Table 2 summarizes the results of multivariate logistic regression analysis for predicting good outcome. The model included age, gender, duration of symptoms, presurgical mJOA score, Babinski’s sign, baseline CMCT for biceps, abductor digiti minimi, tibialis anterior muscles, and length of the lesion on sagittal T2wi MRI. Of the other variables included, statistically significant predictors of better outcomes were shorter length of the lesion on T2wi (OR 0.69; 95% CI 0.55–0.81; *p* < 0.001) and lack of Babinski’s sign (OR 0.15; 95% CI 0.03–0.79; *p* = 0.025).

**Table 2:** Multivariate logistic regression for predictors of good outcome

Predictor	OR	95% CI	*p* value
Gender (male)	1.471	0.324–6.68	0.617
Age (younger)	1.084	0.991–1.185	0.077
Duration of symptoms	1.041	0.8–1.355	0.763
Presurgical mJOA score	0.67	0.401–1.117	0.124
Babinski’s sign	0.146	0.027–0.788	*0.025*
CMCT (biceps)	1.354	0.773–2.372	0.290
CMCT (abductor digiti minimi)	1.234	0.896–1.701	0.198
CMCT (tibialis anterior)	1.003	0.827–1.217	0.972
Length of the lesion on T2wi-MRI	0.668	0.548–0.814	*< 0.001*

CMCT, central motor conduction time; mJOA, modified Japanese Orthopedic Association

**Fig. 2:**
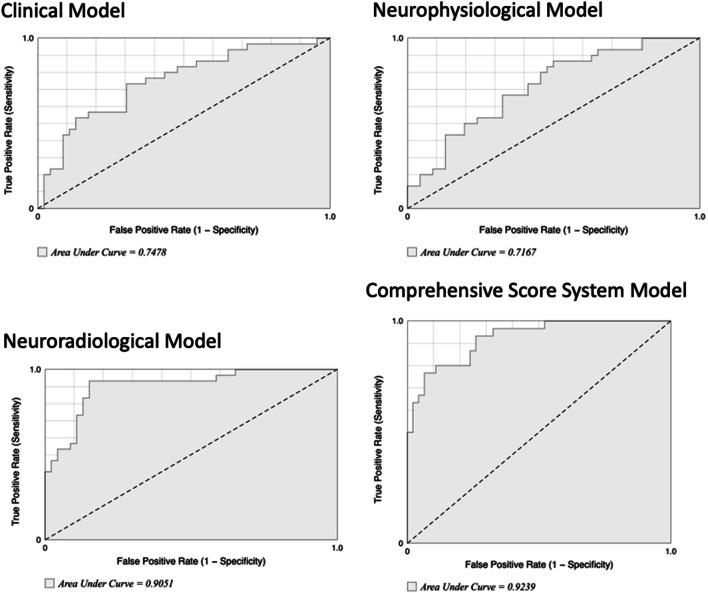
ROC curve analysis of partial and comprehensive models. Figure shows ROC curve analysis of each single model and the comprehensive model. Comprehensive score system showed the best accuracy (AUC: 0.92) compared with each partial model

The analysis of the AUC–ROC curves for good clinical outcome shows that a model made of combining all classes of variables (clinical, neuroradiological, and neurophysiological data) obtained a AUC value of 0.92 showing the best accuracy of CSS compared with other models (Fig. 2).

## Discussion

Cervical myelopathy can cause different clinical pictures varying from no symptom and sign to motor deficits and gait disturbances associated to sphincter dysfunctions [6, 10]. Natural history of CSM is not well characterized [1], but sometimes it may determine a progressive neurological deterioration; however, criteria for patients selection and timing for surgery are controversial [4]. Clinical history of CSM patients often requires a “wait and see period” [5, 12] to evaluate the progression rate of the disease, but no accordance about the best time point for surgery exists [4]. MRI or neurophysiological evaluation by MEP or SEPs may reveal early signs of cervical spinal cord involvement even if neurological evaluation might be still unremarkable [8, 14]. On the other hand, due to time course of the CSM, at some points of the disease, some or all diagnostic exams such as MRI or neurophysiological evaluation may show signs of myelopathy [6, 24].

Because patients with CSM are at increased risks of neurological deterioration or spinal cord injury with nonoperative management and early surgery was linked with better outcomes, there is a trend toward attention to mildly symptomatic CSM [4].

Surgery is beneficial at a certain point of the disease in some patients [4, 15]; however, there is not accordance about timing for surgery [9], and no test is validated to support clinician in decision-making process or in patients selection.

Usually in diagnostic workup of CSM patients, after neurological assessment, neurophysiological and neuroradiological evaluations may be suggested [14, 30], and each of these examinations may give a piece of information about underlying myelopathy, and some diagnostic and treatment algorithms have been developed accordingly [22].

Neurological examination may reveal signs of impairment of central motor pathways [10, 11], and we found a significant negative prognostic value of Babinski’s sign in the multivariate analysis (Table 2).

Neurophysiological assessment either by SEPs [18, 19] or MEP [3] demonstrated to be useful in evaluation of CSM patients in pre- and postsurgical period.

MEP may be a useful neurophysiological tool in diagnostic process [8] by evaluation of CMCT, muscle responses, and interside difference according to previous studies [8] and current guidelines [21]. Moreover, MEP may show an early increase of CMCT of upper limbs when signs of involvement of cervical spinal cord in these muscles are still lacking [8, 14]. Indeed, in CSM patients, clinical signs might not be present in the initial phase of the disease when cervical spinal cord compression may cause only involvement of lower limbs with pure paraparesis [7]. Moreover, the prognostic value of MEP and SEP has also been described [3, 14]. We evaluated CMCT values of both sides for each muscle, because CMCT it is an indirect parameter of the cortico-spinal tract involvement [3, 7].

MRI has a leading role in diagnostic process giving information about level of stenosis and intramedullary lesions [17] but conflicting results raised about increase signal intensity on cervical MRI and its correlation with clinical features and outcomes [17, 26]. We found that the length of spinal cord lesion detected on T2wi-MRI had a significant negative prognostic value in CSM patients. We performed a visual and not a morphometric evaluation of cervical MRI for a number of reasons. Several studies have already performed morphometric analyses about cervical stenosis comparing spinal canal and spinal cord diameters along with CSF signal around the cord or angles [13, 25]. Moreover, due to the highly mobile nature of the cervical spine and the fact that usually most MRIs are obtained only in one single position, dynamic cord compression can be an elusive diagnosis that is often missed and not well-understood [29] especially when stenosis involves several levels. In fact, even if cervical canal is not stenotic on rest cervical MRI, intramedullary lesion on MRI may be discovered [29]; conversely stenotic cervical canal may be not associated with intramedullary high signal confirming a dynamic process at the basis of spondylotic myelopathy [28].

Multivariate analysis did not show a prognostic value of CMCT alone. On the other hand, analysis of the AUC–ROC curves for good clinical outcome shows that a model made of combining all classes of variables obtained the maximum AUC, and the accuracy of the model reaches its best value (Fig. 2). Conversely, a model made of only clinical or only neuroradiological or only neurophysiological variables alone has a small predictive power, confirming the usefulness of the CSS.

We propose a simple, easily assessable and feasible method to help clinicians in evaluating CSM patients especially those in whom surgical approach is not a straightforward decision and a “wait and see” period may be advisable. Moreover, CSS may be a useful tool to assess postsurgical outcome.

This study demonstrates that CSS taking into consideration neurologic, cervical MRI, and MEP results may be a feasible and helpful method to forecast postsurgical outcome and to guide clinical decision in CSM patients candidate to surgery.

Further studies should be performed in subgroups of patients to assess whether, even if surgery could be less effective [15] in term of recovery, it might be beneficial in preventing progression of neurologic impairments.

### Limitations

This study has some limitations: first, the retrospective design and the consequent use of post hoc hypotheses. Then, patients were allocated to surgery at the discretion of the surgeon, so selection bias cannot be excluded, and prospective studies are warranted to confirm these results in a larger population and to evaluate whether CSS may be helpful in managing decision-making process.

## Conclusion

CSS may be a simple, practical, and useful tool to help clinicians in evaluating CSM patients in whom cervical surgery may be a therapeutic option or in whom “wait and see” period is a more advisable option.

## Electronic supplementary material

ESM 1 (XLSX 14 kb)
